# Supplementary material for: Evolutionary food web model based on body masses gives realistic networks with permanent species turnover
Source: Sci Rep. 2015 Jun 4;5:10955. doi: 10.1038/srep10955 (PMC4455292; doi:10.1038/srep10955)
Supplement: Supplementary Information [file srep10955-s1.pdf]

Evolutionary food web model based on body masses gives realistic networks with permanent species turnover
Supplementary material

K.T. Allhoff *, D. Ritterskamp, †,
B.C. Rall, ‡, B. Drossel, §, C. Guill¶

March 27, 2015

Contents

A The main realisations in comparison **2**

B Network visualisations **3**

C Empirical data **4**

D Cutoff for weak links **5**

E Definitions of topological characteristics of food webs **7**

F Community size spectra **8**

References **9**

*Corresponding author: allhoff@fkp.tu-darmstadt.de — Institute for Condensed Matter Physics, Technical University of Darmstadt, Germany

†Institute for Chemistry and Biology of the Marine Environment, Carl von Ossietzky University of Oldenburg, Germany

‡German Centre for Integrative Biodiversity Research (iDiv) Halle-Jena-Leipzig, Germany; Institute of Ecology, Friedrich Schiller University Jena, Germany; Netherlands Institute of Ecology (NIOO-KNAW), Wageningen, The Netherlands; J.F. Blumenbach Institute of Zoology and Anthropology, Georg-August-University Göttingen, Germany

§Institute for Condensed Matter Physics, Technical University of Darmstadt, Germany

¶Institute for Biodiversity and Ecosystem Dynamics, University of Amsterdam, The Netherlands

A The main realisations in comparison

An overview of the time series of the four simulation runs that were used in our article to evaluate the structural properties of the food webs is presented in Fig. S1. Both competition parameters c_{food} and c_{intra} have a strong effect on the diversity of the emerging food webs of our model. Two trends can be observed: First, the stronger the intraspecific competition c_{intra}, the smaller are the population sizes and the more populations can survive on the same amount of energy provided by the resource. Second, the stronger the competition for food c_{food}, the more often can species displace others resulting in rather small networks with fast evolutionary species turnover.

Additional information to these and further simulations with intermediate values of the competition parameters are available on request.

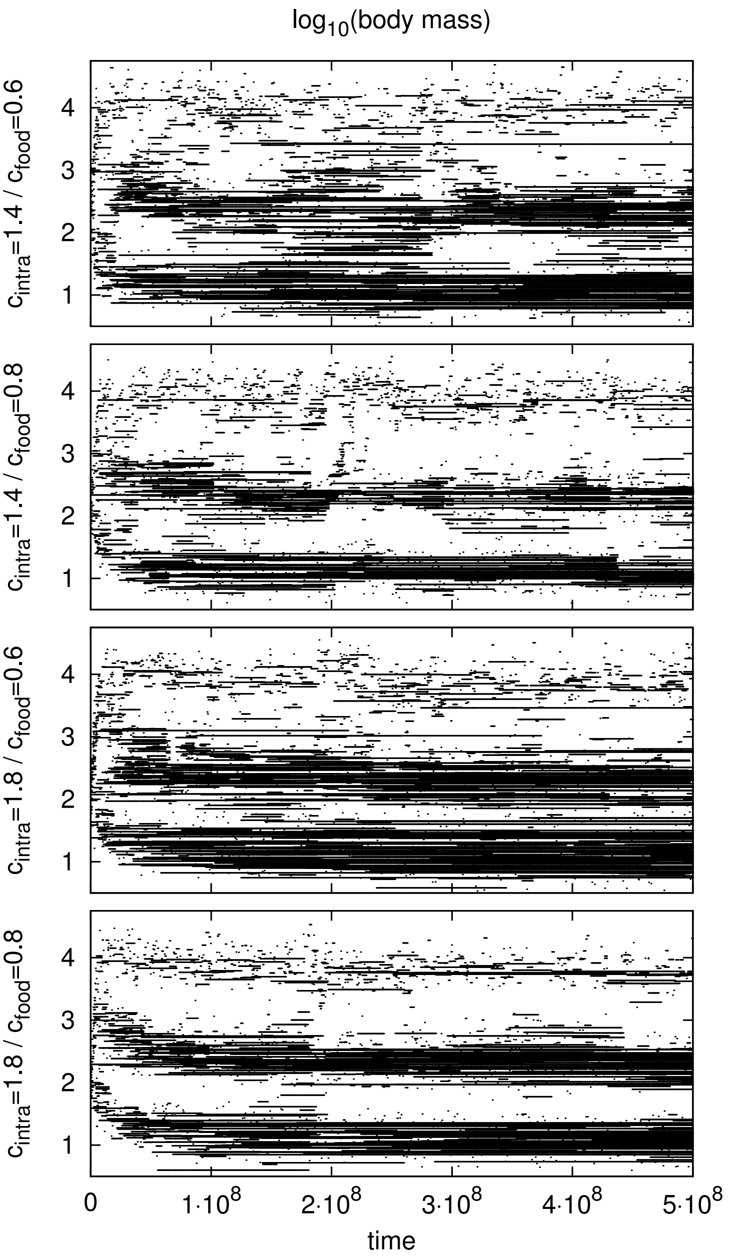

Figure S1: Overview of the time series of the four simulation runs discussed in our article.

B Network visualisations

In Fig. S2, we show four example networks. The food webs correspond to the time points before and after extinction events that are indicated by vertical lines in Fig. 2 of our article. The visualisations were generated using graph-tool (http://graph-tool.skewed.de). A species is represented by a red dot, with the width scaling logarithmically with its biomass density. The vertical position of a species represents its flow-based trophic position, which is the average, weighted trophic position of its prey, plus one. The arrows represent feeding links from a prey species to its predator species. Their width scales logarithmically with the biomass flow.

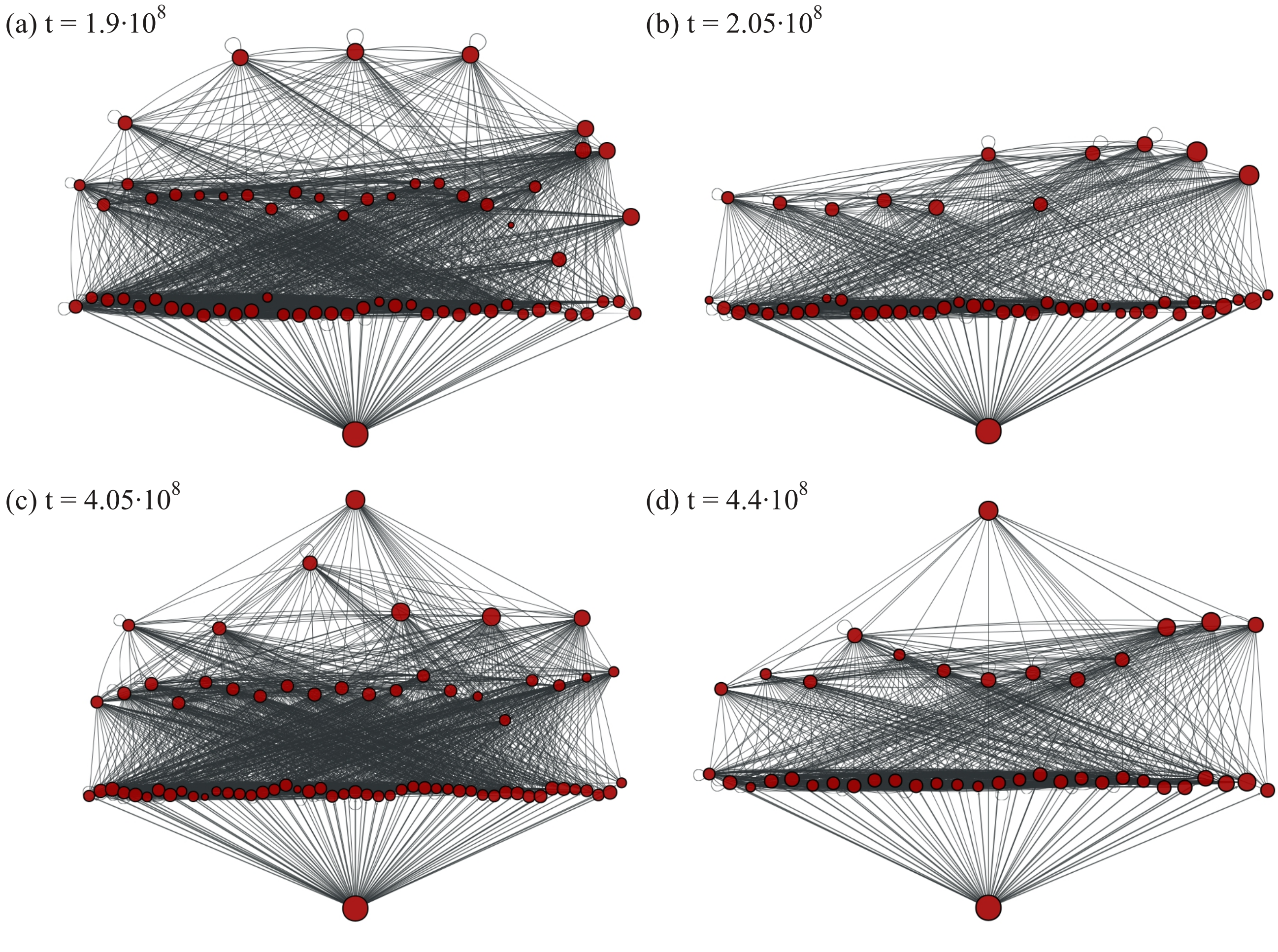

Figure S2: Example food webs for the time points indicated by vertical lines in Fig. 2 of our article. The competition parameters were $c_{\text{intra}} = 1.4$ and $c_{\text{food}} = 0.8$.

C Empirical data

We used a recently compiled collection of empirical food webs from a variety of different ecosystems (estuary, river, lake, marine, terrestrial) to test the evolving model networks against [1] (data provided by Christoph Digel and Jens Riede). This data set contains 65 food webs with species numbers between 27 and 492. We did not evaluate all 65 food webs, but only the 51 networks for which we also had the body masses of all species. The list of empirical food webs used is given in table T2.

Web no.	and name as in [1]	orig. source	Web no.	and name as in [1]	orig. source
3	St. Mark's	[2]	33	Guadeloupe River	[16]
4	Ythan2010	[3]	34	Los Gatos Creek	[16]
6	Alford Lake	[4]	35	Los Trancos Creek	[16]
7	Balsam Lake	[4]	36	San Francisquito Creek	[16]
8	Beaver Lake	[4]	37	Saratoga Creek	[16]
9	Big Hope Lake	[4]	38	Steverson Creek	[16]
10	Bridge Brook Lake	[4]	39	Blackrock	[16]
11	Chub Pond	[4]	41	Ross	[17]
12	Connery Lake	[4]	42	Penetetia Creek	[16]
13	Hoel Lake	[4]	44	Canton	[17]
15	Stink Lake	[4]	45	Dempster	[17]
16	Little Rock Lake	[4]	47	Healy	[17]
17	Sierra Lakes	[5]	50	Stony	[17]
18	Skipwith Pond	[6]	51	Grand Caricaie Cl C1	[18]
19	Tuesday Lake	[7]	52	Coachella	[19]
21	Lough Hyne	[8]	53	EcoWeb 59	[20]
22	Mondego Zostera Meadows	[9]	54	EcoWeb 60	[20]
23	Caribbean Reef, small	[10]	56	Grand Caricaie Sn C2	[18]
25	Weddell Sea	[12, 13, 11]	59	Grand Caricaie Cm M2	[18]
26	Bere Stream	[14]	60	Simberloff_E1	[21]
27	Broadstone Stream	[15]	61	Simberloff_E2	[21]
28	Alamitos Creek	[16]	62	Simberloff_E3	[21]
29	Caldero Creek	[16]	63	Simberloff_E7	[21]
30	Corde Matre Creek	[16]	64	Simberloff_E9	[21]
31	Coyote Creek	[16]	65	Simberloff_ST2	[21]
32	Guadeloupe Creek	[16]			

Table T2: List of empirical food webs.

D Cutoff for weak links

The food-web model defines feeding links between species using Gaussian feeding kernels (see section 2). These feeding kernels extend in principle over the whole niche axis. To obtain meaningful network structures, very weak links have to be cut off. We removed all links that contribute less than a certain fraction of the average link to the total resources of the respective consumer. In this appendix we demonstrate the effects of different cutoff levels (Fig. S3). We also show how the networks generated with the model by Loeuille and Loreau [22] are affected when this cutoff criterion is applied. In general, a lower cutoff value leads to less links being removed and thereby to a higher connectance. This is accompanied by a lower fraction of top species, more species that are a part of feeding loops or that are cannibalistic, and overall more similar species (in terms of decreasing standard deviations of vulnerability and linkedness). Also, more links in the networks increase the clustering coefficient and decrease the characteristic path length.

The networks that were generated with the model of Loeuille and Loreau overall look more realistic if our cutoff criterion is applied instead of the original one. We note for example that with our cutoff criterion, the standard deviations of generality and vulnerability are higher than if the original rule is used (compare Fig. 4 g,h and Fig. S3 g,h). This also means that the entire distributions of generality and vulnerability become broader than those shown in Fig. 3 in the main text. However, they are still not as broad as those produced with our new model or even those observed in the empirical food webs (results not shown).

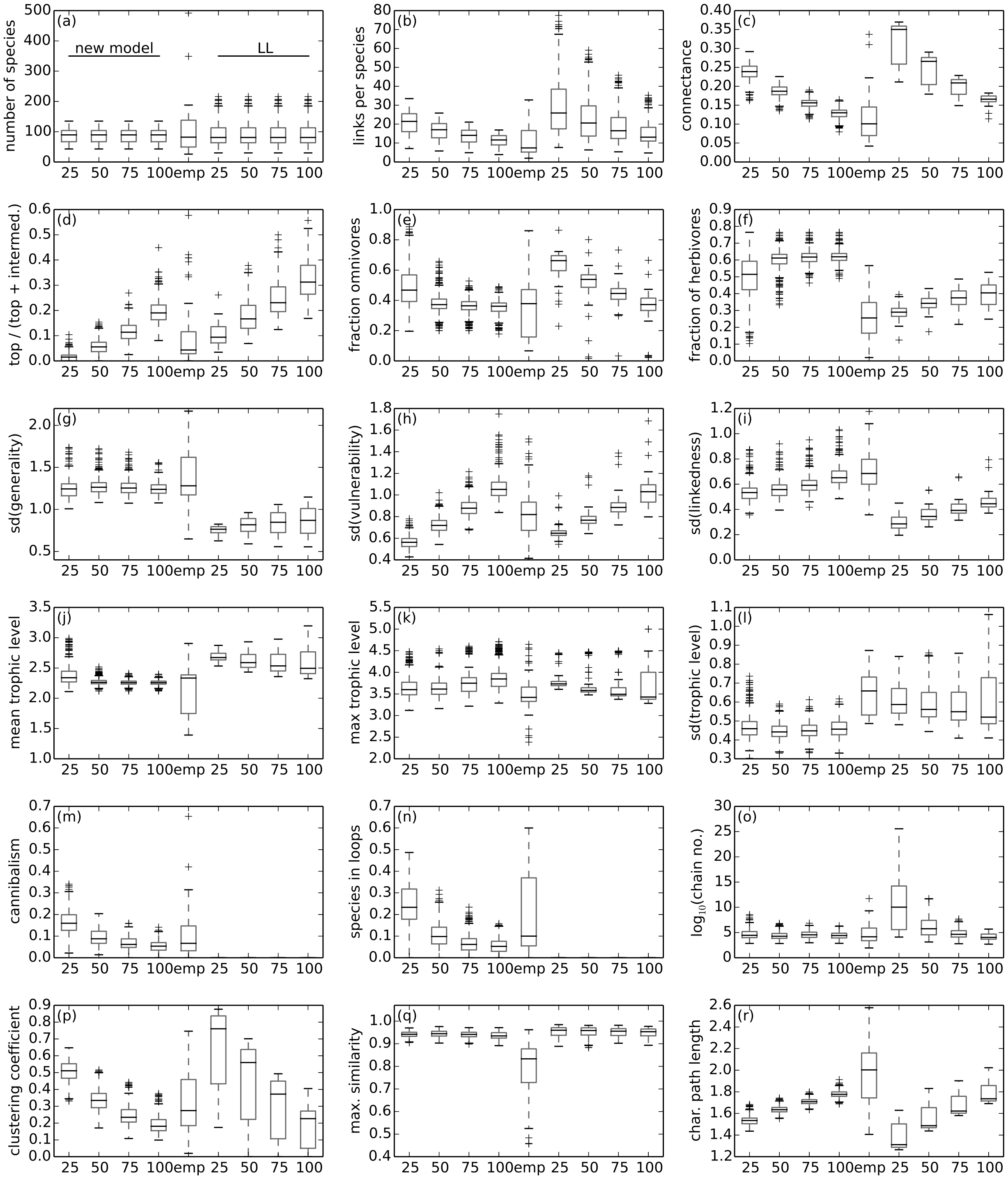

Figure S3: The influence of different cutoff threshold values. A link is regarded as absent if it contributes less than x% of the average link to the total resources of a consumer. The first four entries represent an average over the four main realisations of the new model. **emp**: Average over 51 empirical food webs. **LL**: Average over 75 simulations of the model of Loeuille and Loreau [22].

E Definitions of topological characteristics of food webs

We here summarise the definitions of the 18 topological characteristics we used to evaluate the food-web structure of both model and empirical food webs.

**number of species:**	number of nodes in the network, S
**links per species:**	number of edges, L, divided by number of nodes, S
**connectance:**	number of edges divided by maximum potential number of edges (S^2)
**top:**	number of top species (species without a predator)
**intermediate:**	number of intermediate species (species with both predators and prey)
**fraction omnivores:**	fraction of species with prey from more than one trophic level
**fraction herbivores:**	fraction of species that feeds only on the external resource
**generality:**	distribution of number of prey species for all species, normalised with the average number of links per species
**vulnerability:**	distribution of number of predators for all species, normalised with the average number of links per species
**linkedness:**	distribution of number of prey species plus number of predators for all species, normalised with two times the average number of links per species
**sd():**	standard deviation of a distribution
**mean trophic level:**	mean of the short-weighted trophic levels of all species, cf. [23]
**max. trophic level:**	maximum over the short-weighted trophic levels of all species
**fraction cannibals:**	fraction of species with a cannibalistic link
**fraction species in loops:**	fraction of species that is part of at least one feeding loop, i.e., link patterns of the type i feeds on j, j feeds on k, k feeds on i (excluding cannibalism)
**chain number:**	number of different food chains. To avoid divergence, prior to calculating this number cannibalistic links are removed and feeding loops are cut open by removing links within loops where the predator is on a lower average trophic level than the prey.
**clustering coefficient:**	also: transitivity ratio, cf. [24]. Probability that if species i and j are connected and j and k are connected, i and k are connected, too.
**similarity:**	defined for pairs of species i and j as number of links shared by the species divided by total number of links of the two species. We show the maximum over all pairwise similarities.
**characteristic path length:**	mean of average distance between any two species in the network (measured in feeding links)

F Community size spectra

We computed community size spectra for both models discussed in the manuscript, as shown in Fig. S4. The black dots represent the biomass densities of single species, with the red line representing a linear function obtained by orthogonal regression. The black lines represent the total biomass densities with a resolution of 100 bins. The shape of these curves is robust to bin numbers between 20 and 500.

In panel (a) one clearly recognizes the body mass clusters shown in Fig. 2 in the mansucript and in Fig. S1 in appendix A. The number of species with a rather small body mass is higher than the number of species with a rather big body mass. However, their biomass densities are smaller, leading to a similar amount of total biomass per body mass cluster. Very similar results were observed in empirical data, see Fig.21.1 in [25], and obtained (with a less pronounced clustering) in PDMM communities, see Fig. 22.8 in [25]. The positive slope of the red line is consistent with the energetic equivalence rule, which suggests an exponent of 0.25.

The situation is different in the model by Loeuille and Loreau [22], as shown in panel (b). The number of species per trophic level is approximately the same for all levels and the species on higher trophic levels have smaller population sizes than the species on lower trophic levels. This is due to the fact that all body masses are in the same order of magnitude, meaning that the allometric scaling of the respiration rates can not overcompensate the efficiency losses from one trophic level to the next.

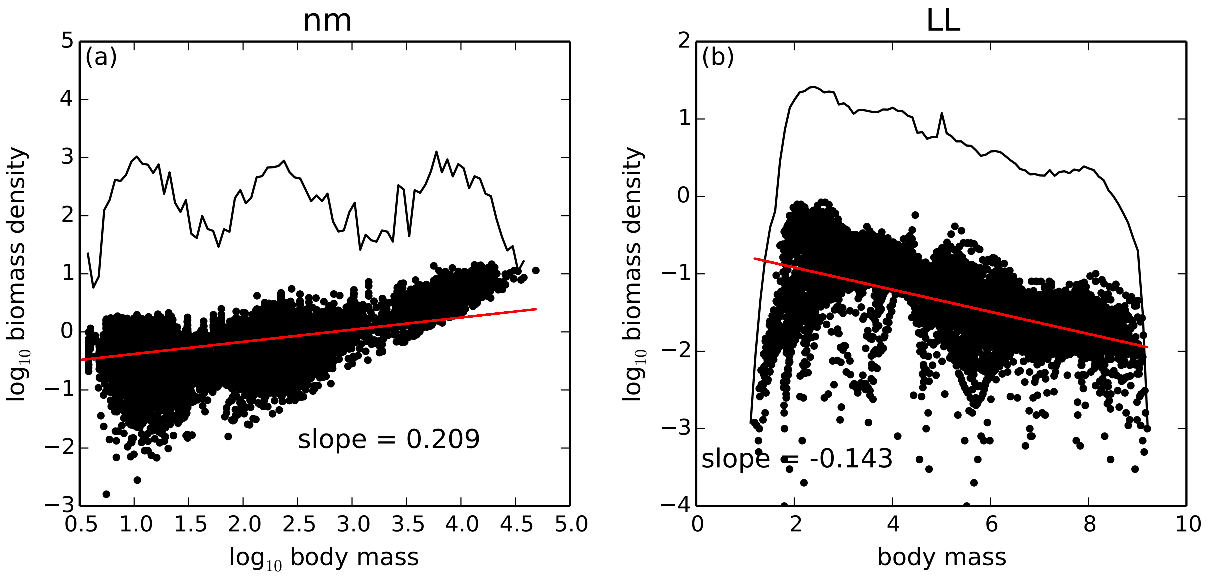

Figure S4: Community size spectra for both models discussed in the manuscript. **nm**: Average over the four main realisations of the new model. **LL**: Average over 75 simulations of the model of Loeuille and Loreau [22].

References

[1] J.O. Riede et al. Adv. Ecol. Res. 42, 139–170 (2010).

[2] Christian, R. & Luczkovich, J. (1999). Organizing and understanding a winter's sea- grass foodweb network through effective trophic levels. Ecol. Model. 117, 99–124.

[3] Cohen, J.E., Schittler, D.N., Raffaelli, D.G., and Reuman, D.C. (2009). Food webs are more than the sum of their tritrophic parts. Proc. Natl. Acad. Sci. USA 106, 22335–22340.

[4] Havens, K. (1992). Scale and structure in natural food webs. Science 257, 1107–1109.

[5] Harper-Smith, S., Berlow, E., Knapp, R., Williams, R., and Martinez, N. (2005). Communicating ecology through food webs: Visualizing and quantifying the effects of stocking alpine lakes with fish. In: Dynamic Food Webs: Multispecies assemblages, ecosystem development, and environmental change. Elsevier/Academic Press.

[6] Warren, P.H. (1989). Spatial and temporal variation in the structure of a fresh-water food web. Oikos 55, 299–311.

[7] Jonsson, T., Cohen, J.E., and Carpenter, S.R. (2005). Food webs, body size, and species abundance in ecological community description. Adv. Ecol. Res. 36, 1–84.

[8] U. Jacob, unpublished data

[9] Patricio, J., and Marques, J.C. (2006). Mass balanced models of the food web in three areas along a gradient of eutrophication symptoms in the south arm of the Mondego estuary (Portugal). Ecol. Model. 197, 21–34.

[10] Opitz, S. (1996). Trophic interactions in Caribbean coral reefs. Technical Report 43. ICLARM, Manily.

[11] Jacob, U. (2005). Trophic Dynamics of Antarctic Shelf Ecosystems – Food Webs and Energy Flow Budgets. Thesis, University of Bremen.

[12] Brose, U., Jonsson, T., Berlow, E.L., Warren, P., Banasek-Richter, C., Bersier, L.F., Blanchard, J.L., Brey, T., Carpenter, S.R., Cattin Blandenier, M.-F., Cushing, L., Dawah, H.A., et al. (2006a). Consumer-resource body-size relationships in natural food webs. Ecology 87, 2411–2417.

[13] Brose, U., Williams, R.J., and Martinez, N.D. (2006b). Allometric scaling enhances stability in complex food webs. Ecol. Lett. 9, 1228–1236.

[14] Woodward, G., Papantoniou, G., Edwards, F., and Lauridsen, R.B. (2008). Trophic trickles and cascades in a complex food web: Impacts of a keystone predator on stream community structure and ecosystem processes. Oikos 117, 683–692.

[15] Woodward, G., Speirs, D.C., and Hildrew, A.G. (2005). Quantification and temporal resolution of a complex size-structured food web. Adv. Ecol. Res. 36, 85–135.

[16] Harrison, K. (2003). Effects of Land Use and Dams on Stream Food Web Ecology in Santa Clara Valley, California 127. Thesis, San Francisco State University.

[17] Townsend, C.R., Thompson, R.M., McIntosh, A.R., Kilroy, C., Edwards, E., and Scarsbrook, M.R. (1998). Disturbance, resource supply, and food-web architecture in streams. Ecol. Lett. 1, 200–209.

[18] Cattin Blandenier, M.-F. (2004). Food Web Ecology: Models and Application to Conservation. Thesis, Université de Neuchâtel (Suisse).

[19] Polis, G.A. (1991). Complex trophic interactions in deserts: An empirical critique of food-web theory. Am. Nat. 138, 123–155.

[20] Cohen, J.E. (1989). Ecologists Co-operative Web Bank (ECOWebTM).

[21] Simberloff, D.S., and Abele, L.G. (1975). Island biogeography theory and conserva- tion practice. Science 191, 285–286.

[22] Loeuille, N. and Loreau, M. (2005). Evolutionary emergence of size-structured food webs. Proc. Natl. Acad. Sci. USA, 102: 5761–5766.

[23] Williams, R.J. and Martinez, N.D. (2004). Limits to trophic levels and omnivory in complex food webs: theory and data. Am. Nat. 163, 458–468.

[24] Newman, M.E.J. (2003). The structure and function of complex networks. SIAM review 45, 167–256.

[25] Axel G. Rossberg. Food webs and biodiversity: foundations, models, data. John Wiley & Sons, ISBN 9-780470973-55-4.
